# Vaccination Rate and Incidence of COVID-19 and Case Fatality Rate (CFR): A Correlational Study Using Data From 2019 to 2021

**DOI:** 10.7759/cureus.28210

**Published:** 2022-08-20

**Authors:** Jayakumary Muttappallymyalil, Satish Chandrasekhar Nair, Ramadas Changerath, Anusha Sreejith, Sashank Manda, Jayadevan Sreedharan

**Affiliations:** 1 Community Medicine, Gulf Medical University, Ajman, ARE; 2 Medical Affairs, Tawam Hospital, Al Ain, ARE; 3 Institutional Research, Khalifa University, Abu Dhabi, ARE; 4 Medicine, Manipal Academy of Higher Education, Mangalore, IND

**Keywords:** correlation, vaccination rate, cfr, covid-19, high-income countries, low-income countries, bi-weekly incidence rate

## Abstract

Introduction: COVID-19 has infected over 300 million people and killed almost five million people worldwide. The rapid development and deployment of vaccines almost a year after the initial outbreak was poised to contain the pandemic and enable the mobilization of the people and the economy. Vaccine deployment and containment of the pandemic have been far from uniform across the world. There is a lack of a clear understanding of the correlation between the COVID-19 vaccination rates and the incidence of the COVID-19 disease and COVID-19 mortality.

Aim: The study aims to determine the correlation between the COVID-19 vaccination rate and the bi-weekly incidence rate of the COVID-19 disease to better understand the correlation between the vaccination rate and the COVID-19-related fatality in various countries.

Materials and methods: Data from vaccination and the case fatality rate were abstracted until September 15, 2021, and from October 15 to October 31, respectively, for the various countries categorized based on their income levels. The bi-weekly COVID-19 incidence rate per million population and the case fatality rate was analyzed using SPSS version 27 (IBM Corp., Armonk, NY), followed by frequencies and percentages. Spearman rank correlation was used to determine the relationship between the variables.

Results: A total of 191 countries were included in the study. The vaccination rate ranged from 0.03% to 82.1%, CFR from 0.14% to 32.1%, and the bi-weekly incidence rate ranged from zero to 1,283 per million population. A positive correlation was observed between vaccination rate and bi-weekly incidence rate (+0.57), whereas a negative correlation was observed between vaccination rate and CFR (-0.34). The results indicate a moderate positive correlation between vaccination rate and bi-weekly incidence rate and a weak negative correlation between vaccination rate and case fatality rate.

Discussion and conclusion: Our study is interesting for the observation that the bi-weekly incidence rate of COVID-19 positively correlated with the rate of vaccination. In contrast, the vaccination rate correlated negatively with the case fatality rate. Although several factors may have contributed to the increased incidence rates for COVID-19, these observations refute the myth that COVID-19 vaccination offers complete protection from reinfection, especially in the backdrop of easing pandemic containment measures by some countries. An increase in the vaccination rate is certainly a positive contributor to the decreasing case fatality observed.

## Introduction

The eruption of the novel coronavirus (SARS-CoV-2) from Wuhan in China in late December 2019 set a cascade of events that resulted in the destruction of world economies and the collapse of the public health system in many countries [1,2]. The virus has infected over 300 million people and killed almost five million worldwide [3]. The various governments enforced nationwide lock-downs, travel bans, and the curb on huge gatherings to contain the pandemic [4]. Personal and community sanitization, social etiquette, masks, and physical distancing measures were implemented to prevent the virus from spreading. Trace, quarantine, isolate and treat protocols replaced the traditional guidelines to manage the pandemic [5]. Rapid development and deployment of vaccines against the virus became the only options to provide individual and population-level immunity. Undoubtedly, vaccines' rapid development was critical for resuming routine global social and economic activities [6]. To facilitate the development and dissemination of the SARS-CoV-2 vaccines, the US government committed over 10 billion dollars, intending to deliver almost 300 million doses in the first quarter of 2021 [7]. Following the development of vaccines, the challenges for vaccine uptake and vast acceptance were also anticipated [8]. Therefore, a 23-person Working Group on Readying Populations for COVID-19 Vaccines was formed to synthesize the opportunities and challenges for effective vaccine deployment [9,10].

In less than a year since the onset of the COVID-19 pandemic, several safe and effective vaccine candidates were successfully tested and made available for public emergency use by the World Health Organization [11]. To ensure the incidence of COVID-19 is reduced rapidly, the equitable deployment of the COVID-19 vaccine was critical but not without challenges [12]. COVID-19 has disproportionally affected economically and socially disadvantaged people. Several reports highlight the disparity in vaccine deployment and the subsequent hardships certain countries face, especially countries with low socioeconomic order [11]. Research and statistics indicate that almost 25.3% of the world population has received at least one dose of a COVID-19 vaccine, although 3.44 billion doses have been administered globally, and 29.6 million are now administered each day [12]. On the contrary, approximately 1% of people in low-income countries have received at least one dose of the vaccine [13]. 

Very few studies have addressed the correlation between vaccination status and new COVID-19 cases. An increase in the vaccination coverage was found to be negatively correlated to new cases and ICU patients per million population. However, vaccines benefitted in the prevention of severity and transmission of infection [14]. There was no significant reduction in COVID-19 cases as per the ascent in the vaccination processes according to a study conducted in 68 countries and the US [15]. But in the US, a reduction in mortality and case incidence was reported due to the increase in vaccination coverage even during the time of both alpha and delta variants [16].

There is a lack of a clear understanding of the correlation between the COVID-19 vaccination rates and the incidence of the COVID-19 disease. Therefore, this study's novelty and purpose are to ascertain the correlation between the COVID-19 vaccination rate and the incidence of COVID-19 in high-income, low, and lower-middle-income countries. An additional objective of the study was to assess the correlation between the vaccination rate and the COVID-19 case fatality rate in various countries.

## Materials and methods

A correlational study was adopted for the current study. COVID-19 specific data and other related information for the various countries, such as the country-specific vaccination coverage, number of new cases, and the number of new deaths, were collected. The ourworldindata.org data resource listed each country profile with interactive visualizations, explanations of the presented data, and details of the data sources [17]. Country-specific data was retrieved and recorded using the “country name” as the “keyword.” From a total of 207 countries listed, data from 191 countries were harvested after meeting the criteria for the completeness of data. Vaccination data until September 15, 2021 and the case fatality and new COVID-19 cases were extracted from October 15 to October 31, 2021. The various countries were categorized as low, lower-middle, upper-middle, and high-income, based on their income levels as per the World Bank [18]. The number of bi-weekly new COVID-19 cases per million population and the CFR, defined as the ratio between confirmed deaths and confirmed cases, were calculated [19]. This study was approved by the Institutional Review Board of Gulf Medical University, Ajman, United Arab Emirates.

Data analysis

Data extracted were analyzed using SPSS version 27 (IBM Corp., Armonk, NY) and represented as frequencies and percentages, as shown in the tables and figures. Spearman correlation coefficient was used to determine the relationship between the variables.

## Results

**Figure 1:**
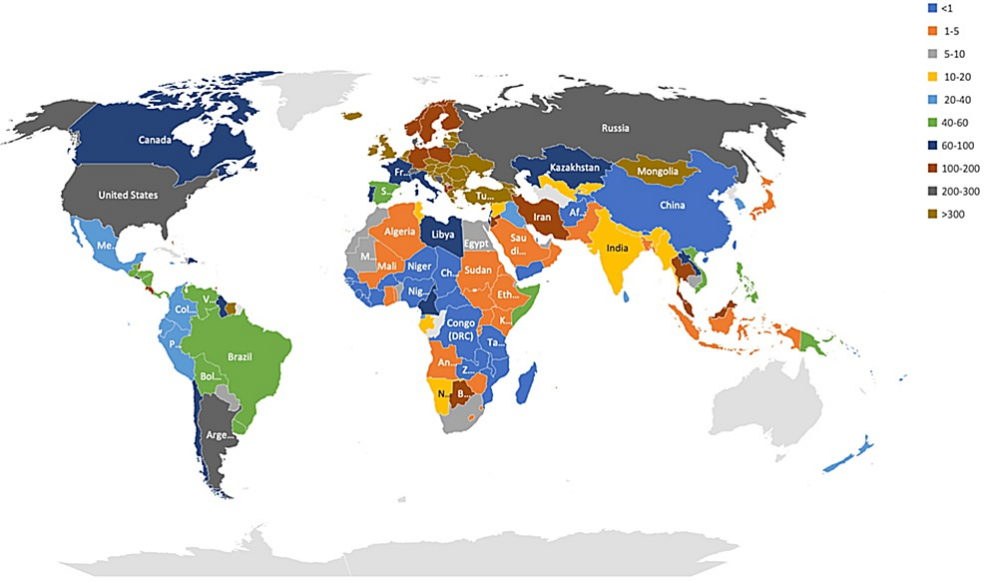
Distribution of bi-weekly average COVID-19 cases/100,000

**Figure 2:**
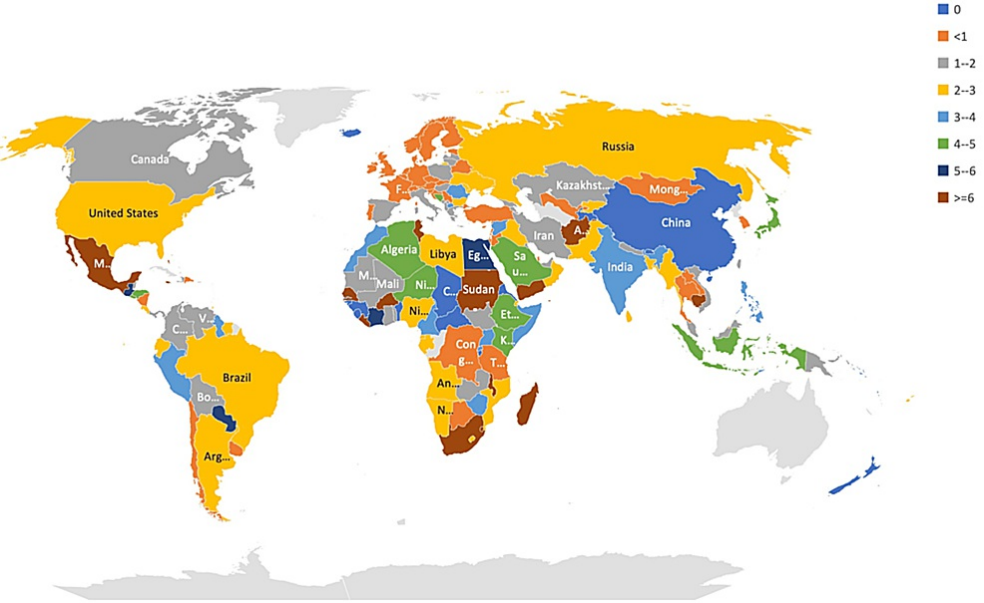
Distribution of case fatality in percentage

**Figure 3:**
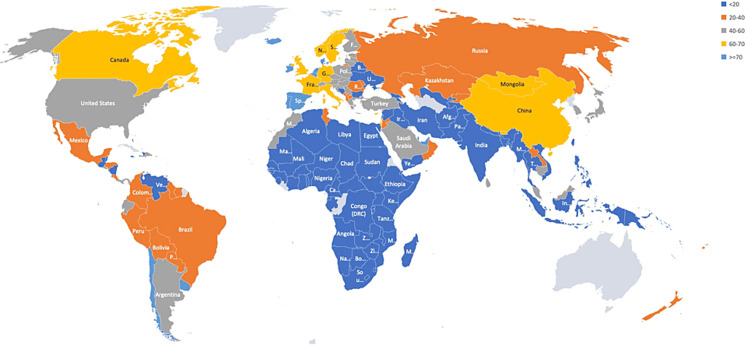
Distribution of vaccination rate in percentage

**Figure 4:**
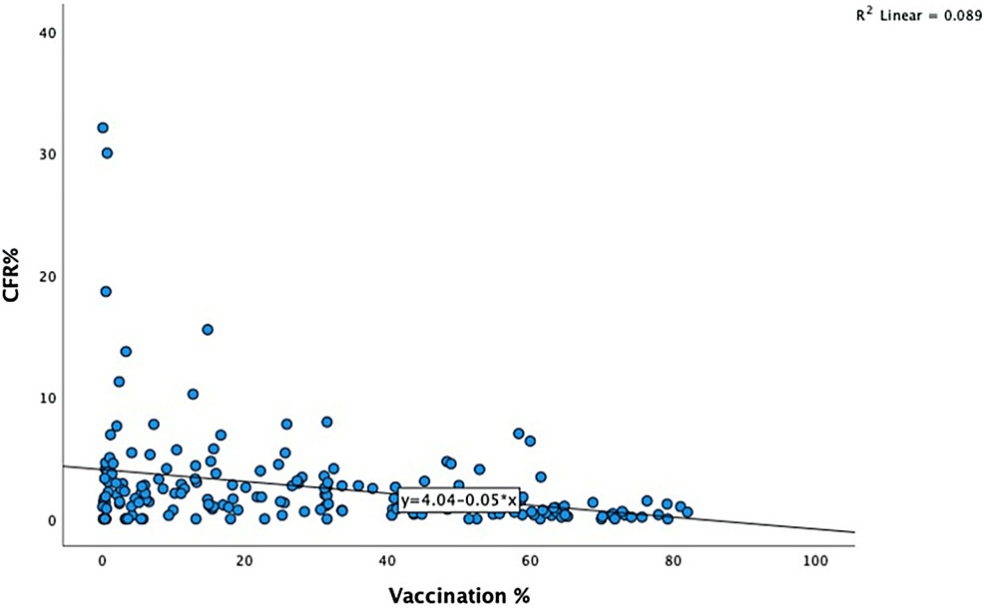
Scatter diagram showing the correlation between vaccination rate and case fatality rate (%)

**Figure 5:**
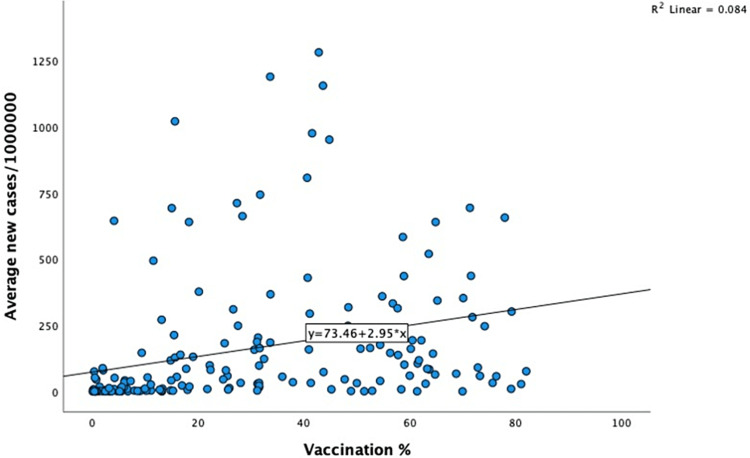
Scatter diagram showing the correlation between vaccination rate and Bi-weekly average COVID-19 cases

Data such as the vaccination rate, bi-weekly case incidence, bi-weekly number of deaths, and the population of the various countries were extracted from the dataset, and the CFR and other outcomes were calculated. A total of 191 countries were included in the study. Vaccination rate data was not available for the ten countries. Interestingly, the vaccination rate varied among countries studied and ranged between 0.03% and 82.1%, and the bi-weekly incidence rate ranged between less than one and 1282/million population (Figure 1). The lowest and the highest range for the CFR observed were 0.14% and 32.1%, respectively (Figure 2). Comparing the vaccination rates across the countries studied indicated that the vast majority (46.4%) had a vaccination rate of less than 20%. On the other spectrum, a 70% or more vaccination rate was noted in 7.7% of the countries. Almost 16.5% of the countries vaccinated at least 60% of their population (Figure 3). A similar pattern of case incidence rate was noted. The number of COVID-19 cases per million ranged between less than one and 300 across the countries studied (Table 1, Figures 1-3). Concerning the CFR, 46% of the countries studied indicated a CFR of less than two, and less than 10% (n=16, 8.4%) of the countries reported a CFR of greater than or equal to six. Interestingly, 13.6% (n=26) countries reported no CFR during this period. Countries with low and lower middle income demonstrated very low vaccination rates. The vaccination rate in the low-income countries remained low at 20% or less. Approximately 15% of the total 49 countries in the lower-middle-income group showed vaccination rates over 40% (Table 2). In contrast, the vaccination rate recorded for the high-income countries was 84% (47/56) for having vaccinated at least 40% of their population (Table 2). Less than 20% (17%, 8/46) of the upper-middle income countries vaccinated at least 40% of their population. Although marginal, the vaccination rate is shown to have a positive correlation with the CFR for low-income (0.01) and lower-middle countries (0.11) (Table 3). In contrast, the upper-middle-income and high-income countries demonstrated a negative correlation between the vaccination rate and the CFR (Table 3). Overall, for all countries, regardless of their income status, the correlation was positive between bi-weekly new cases per million (0.57) and negative for the CFR (-0.34) (Figures 4, 5).

**Table 1:** Vaccination rate, bi-weekly incidence rate and case fatality rate

	Group	Frequency	Percent
Vaccination rate (%)	<20	84	46.4
	20-40	33	18.2
	40-60	34	18.8
	60-70	16	8.8
	>=70	14	7.7
Bi-weekly incidence rate (per Million)	<1	33	17.3
	1-5	23	12.0
	5-10	13	6.8
	10-20	10	5.2
	20-40	15	7.9
	40-60	14	7.3
	60-100	15	7.9
	100-200	25	13.1
	200-300	11	5.8
	>=300	32	16.8
CFR (%)	0	26	13.6
	<1	45	23.6
	1-2	43	22.5
	2-3	28	14.7
	3-4	15	7.9
	4-5	12	6.3
	5-6	6	3.1
	>=6	16	8.4

**Table 2:** Vaccination rate in countries with different levels of income

Vaccination rate (%)	Group	Income Level				
		Low	Lower- middle	Upper-middle	High	Total
<20	No.	24	36.0	20.0	3.0	83.0
	%	100	73.5	43.5	5.4	47.4
20-40	No.	--	6.0	18.0	6.0	30.0
	%	--	12.2	39.1	10.7	17.1
40-60	No.	--	4.0	6.0	22.0	32.0
	%	--	8.2	13.0	39.3	18.3
60-70	No.	--	2.0	2.0	12.0	16.0
	%	--	4.1	4.3	21.4	9.1
>=70	No.	--	1.0	--	13.0	14.0
	%	--	2.0	--	23.2	8.0

**Table 3:** Correlation between vaccination rate and, bi-weekly cases per million and CFR%

Income Level	Correlation coefficient with Vaccination rate	
	Bi-weekly new cases/1000000	CFR%
Low	-0.07	0.01
Lower-middle	0.44	0.11
Upper-middle	0.12	-0.25
High income	-0.07	-0.37
Overall	0.57	-0.34

## Discussion

Regardless of the speed at which COVID-19 vaccines were developed and approved for emergency use by the World Health Organization, COVID-19 infection continues to disrupt and displace human lives [20,21]. The vaccines were deployed in several phases to facilitate availability for the most vulnerable population in several countries, such as the healthcare providers and the elderly. The lack of a standard for the effective deployment of vaccines across the globe, and concerns surrounding the safety and efficacy of the COVID-19 vaccines, generated abundant variability in the general acceptance and the willingness to be vaccinated, thus affecting vaccination rates [22]. Our data showed that a vast majority of the countries (61%) had vaccinated only 20% of the population. This is despite the emergency approval for several COVID-19 vaccines by the World Health Authorization. Almost 22.5% of the countries showed a higher vaccination rate, and more than 60% of the total population in these countries were vaccinated (Table 1). Several factors have contributed to the overwhelming disparity in the vaccination rates across countries. Vaccination inequity results from the lack of a) effective global partnerships, b) vaccine supply chain logistics, c) vaccine apathy by the population and d) proactive strategies by the government [22]. It is no surprise that the bi-weekly COVID-19 incidence varied between less than one and 300 per million population across the various countries (Table 1). Excluding the challenges related to poor testing and underreporting, the effective containment of COVID-19 is dependent on each country’s strategic preparedness and response plan as the World Health Organization prescribes [23]. The predominant variables for the preparedness to prevent CFR are detection, healthcare-seeking behavior, rapid reporting, health system, compliance with international norms, and the risk environment [23].

The upper-middle-income and high-income countries demonstrated a negative correlation between bi-weekly new cases per million and the CFR (Table 3). The results demonstrate that the bi-weekly new cases per million were positively correlated with the CFR for the low-income (0.014) and lower-middle countries (0.11) (Table 3). COVID-19 has disproportionately affected minority groups and countries facing economic and social barriers to healthcare. Countries on a low socioeconomic scale, with a higher population density, poverty, and congested housing conditions have also been reported to have low vaccination rates [24]. Caspi et al. recently demonstrated that a lower COVID-19 vaccination percentage was associated with lower socioeconomic status and a high active disease burden in Israel [24]. Further, it was found that lower socioeconomic status impacted a higher disease burden because the at-risk population was not vaccinated at the targeted rates [24]. The low vaccination rates in low-income countries can be partly explained by the limited accessibility and availability of healthcare resources.

It is paradoxical that in several high-income countries, despite the COVID-19 vaccination rate advancement, there continues to be a rise in the number of new cases per million. The emergence of the newer COVID-19 infectious strains, the average age of the population, the comorbidity index of the population, herd immunity, and other social and political factors are potential contributors to the observed anomaly [25]. Poor vaccination acceptance by the population is also a significant hurdle to decreasing vaccination rates [26]. The positive correlation observed in the study between the vaccination rate, and the COVID-19 disease incidence rate refutes the myth that vaccination offers complete protection from reinfection for COVID-19 [27], especially in the light of easing pandemic containment measures by certain countries. An increase in the vaccination rate is certainly a positive contributor to the observed decreasing case fatality rate.

## Conclusions

The vaccination rate ranged between 0.03% and 82.1%; the bi-weekly incidence rate ranged between <1 and 1,282/million population and the CFR ranged between 0.14% and 32.1%. The vaccination rate was positively correlated with the case fatality rate for low-income and low-middle-income countries while negatively correlated with upper-middle-income and high-income countries. Low-income and high-income countries showed a weak negative correlation between vaccination rate and bi-weekly new cases, whereas the middle-income countries showed a positive correlation. Overall, for all countries, the correlation was positive between bi-weekly new cases per million (0.57) and negative for the CFR (-0.34). Although several factors may have contributed to the increased incidence rates for COVID-19, essentially, these observations refute the myth that COVID-19 vaccination offers complete protection from reinfection.
